# Ventilator Dependence Risk Score for the Prediction of Prolonged Mechanical Ventilation in Patients Who Survive Sepsis/Septic Shock with Respiratory Failure

**DOI:** 10.1038/s41598-018-24028-4

**Published:** 2018-04-04

**Authors:** Ya-Chun Chang, Kuo-Tung Huang, Yu-Mu Chen, Chin-Chou Wang, Yi-Hsi Wang, Chia-Cheng Tseng, Meng-Chih Lin, Wen-Feng Fang

**Affiliations:** 1 Division of Pulmonary and Critical Care Medicine, Department of Internal Medicine, Kaohsiung Chang Gung Memorial Hospital, Chang Gung University College of Medicine, Kaohsiung, Taiwan; 2 Graduate Institute of Clinical Medical Sciences, Chang Gung University, Taoyuan, Taiwan; 3 Department of Respiratory Care, Chang Gung University of Science and Technology, Chiayi, Taiwan; 4 Department of Respiratory Therapy, Kaohsiung Chang Gung Memorial Hospital, Chang Gung University College of Medicine, Kaohsiung, Taiwan

## Abstract

We intended to develop a scoring system to predict mechanical ventilator dependence in patients who survive sepsis/septic shock with respiratory failure. This study evaluated 251 adult patients in medical intensive care units (ICUs) between August 2013 to October 2015, who had survived for over 21 days and received aggressive treatment. The risk factors for ventilator dependence were determined. We then constructed a ventilator dependence (VD) risk score using the identified risk factors. The ventilator dependence risk score was calculated as the sum of the following four variables after being adjusted by proportion to the beta coefficient. We assigned a history of previous stroke, a score of one point, platelet count less than 150,000/μL a score of one point, pH value less than 7.35 a score of two points, and the fraction of inspired oxygen on admission day 7 over 39% as two points. The area under the curve in the derivation group was 0.725 (p < 0.001). We then applied the VD risk score for validation on 175 patients. The area under the curve in the validation group was 0.658 (p = 0.001). VD risk score could be applied to predict prolonged mechanical ventilation in patients who survive sepsis/septic shock.

## Introduction

Sepsis is one of the most common reasons for admission to the intensive care unit (ICU) and involves multiple organ dysfunctions, including respiratory failure^1^. It is a costly condition with high morbidity and mortality^2–4^. Sepsis is among the leading burdens of critical illnesses worldwide^5–8^.

After implementation of the recommendations from the “Surviving Sepsis Campaign: International guidelines for the management of severe sepsis and septic shock,” the survival rate of patients with sepsis has improved^9,10^. Despite the improved survival rate, a proportion of them subsequently require mechanical ventilation and ICU admission. Amongst these subsequently ventilated patients, some ventilated patients are weaned off ventilation in one attempt, whereas for others, multiple attempts are required to wean them off the mechanical ventilation successfully. Another proportion of surviving patients may experience repeated failed attempts at being weaned off ventilation. These patients are at a substantial risk of long-term ventilator-dependence.

The Centers for Medicare and Medicaid Services in the United States defines prolonged mechanical ventilation (PMV) as more than 21 days of mechanical ventilation for at least six hours per day^11,12^. It is estimated that between 7,250 and 11,400 patients undergo prolonged mechanical ventilation annually and between 4 and 13 percent of mechanically ventilated patients require prolonged mechanical ventilation^12,13^. The proportion of patients requiring prolonged ventilation varies in different situations^14–18^. Little is known about the proportion of mechanical ventilation-dependent patients who survive sepsis and septic shock. Long-term ventilator use imposes a financial burden on patients, families, and the healthcare facility. Furthermore, it decreases the patient’s quality of life.

We sought to determine the risk factors for ventilator dependence in patients who survive sepsis and septic shock.

## Results

### Patient characteristics and findings

**Figure 1:**
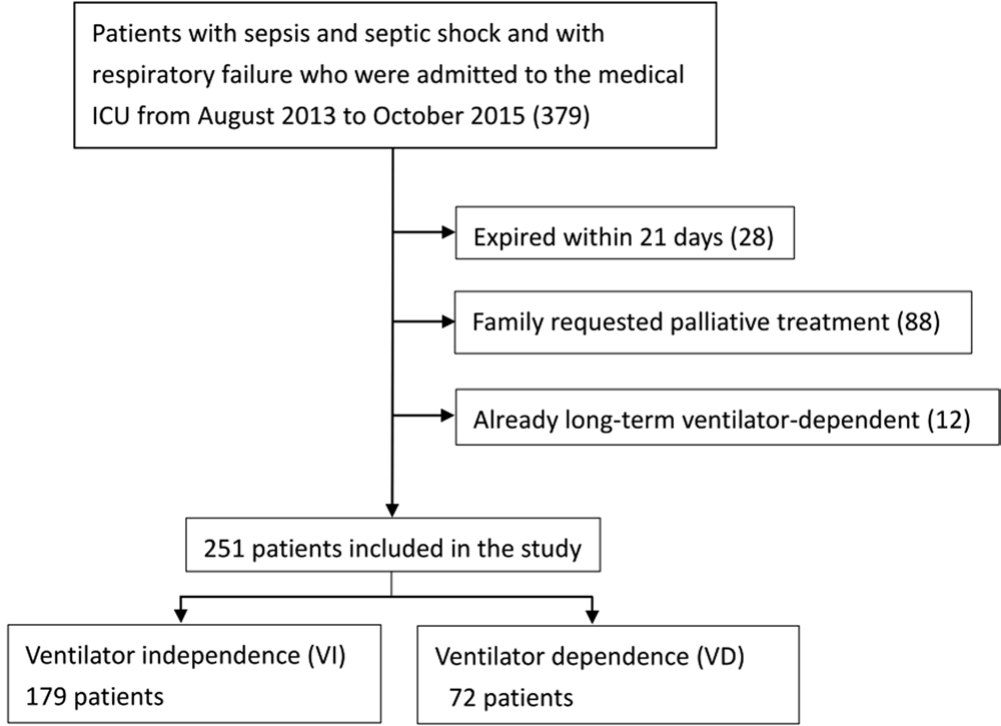
Of the 379 patients who were diagnosed with sepsis/septic shock with respiratory failure between August 2013 and October 2015, 251 patients were included in the final analysis.

**Figure 2:**
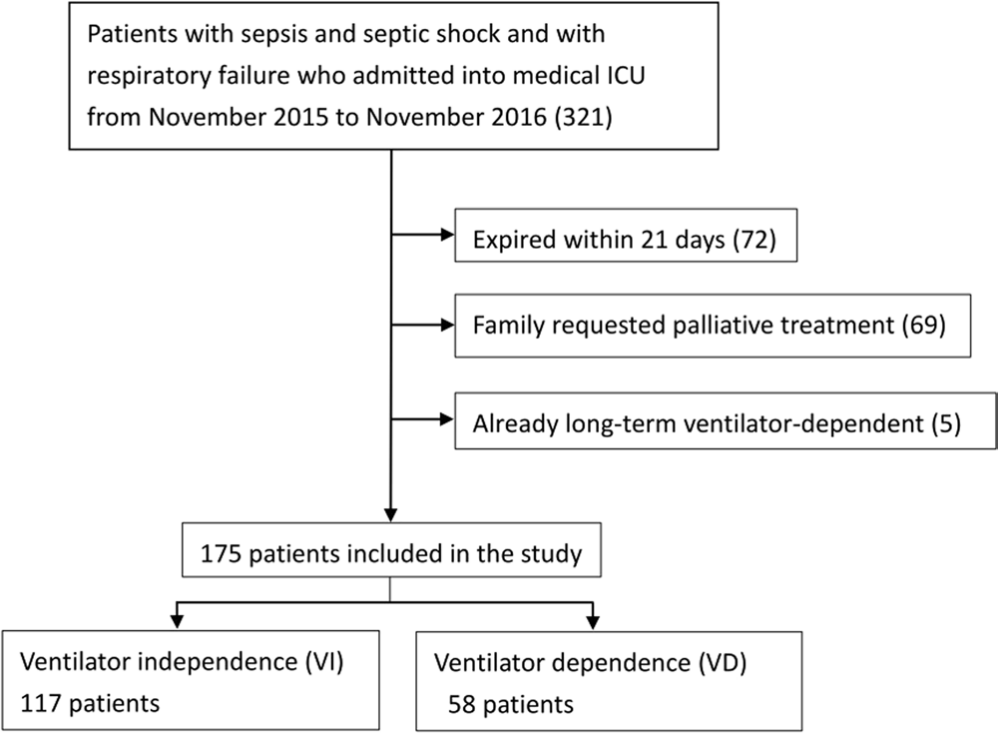
Of the 321 patients who were diagnosed with sepsis/septic shock and respiratory failure between November 2015 and November 2016, 175 patients were included for further analysis as the validation group.

A total of 379 patients with sepsis or septic shock and acute respiratory failure requiring mechanical ventilation were admitted to the medical intensive care unit in the Kaohsiung Chang Gung Memorial Hospital from August 2013 to October 2015. Of the 379 patients, 251 patients were enrolled in the study (Fig. 1). For validation, we collected data from sepsis/septic shock patients with mechanical ventilation admitted to the medical ICU from November 2015 to November 2016 and 175 patients were included in the study (Fig. 2).

Among the 251 patients studied, 69.3% were admitted from the emergency room. The site of suspected infection was of pulmonary origin in 60.6% (Table 1). 72 patients (29%) were ventilator-dependent (on the ventilator for at least 21 days), and 179 (71%) patients were ventilator-independent (needing ventilator support for less than 21 days). The mean age (standard deviation (SD)) of ventilator-dependent patients was older (64.59 (15.67) vs. 69.40 (15.65), *p* < 0.05) (Table 2). No statistical differences were observed in the APACHE II score (*p* = 0.664) or the initial SOFA score (*p* = 0.184) between the ventilator-dependent and ventilator-independent groups. However, there was a significant difference between the ventilator-dependent and ventilator-independent groups regarding previous stroke (*p* = 0.027 in the univariate analysis) (Table 2).

**Table 1:** Patient characteristics and outcome in 251 studied patients.

Patient characteristics	Total (N = 251)	Ventilator-dependent (N = 72)	Ventilator-independent (N = 179)	p-value
Admission source	No. (%)	No. (%)	No. (%)	
Emergency room	174 (69.3)	41 (56.9)	133 (74.3)	0.007
Ward	37 (14.7)	17 (23.6)	20 (11.2)	0.012
Outside ICU	1 (0.4)	1 (1.4)	0 (0)	0.114
Outside hospital	28 (11.2)	12 (16.7)	16 (8.9)	0.079
OPD	4 (1.6)	1 (1.4)	3 (1.7)	0.870
Nursing home	7 (2.8)	0 (0)	7 (3.9)	0.089
Site of suspected infection	No. (%)	No. (%)	No. (%)	
Pulmonary	152 (60.6)	50 (69.4)	102 (57.0)	0.068
Intra-abdominal	17 (6.8)	5 (6.9)	12 (6.7)	0.945
Urinary tract	71 (28.3)	20 (27.8)	51 (28.5)	0.910
Bacteremia	20 (8.0)	4 (5.6)	16 (8.9)	0.371
Skin or soft tissue	21 (8.4)	5 (6.9)	16 (8.9)	0.606
Unidentified infection	15 (6.0)	3 (4.2)	12 (6.7)	0.443
ICU stay	13.12 ± 8.61	21.49 ± 10.67	9.75 ± 4.35	<0.001
Total ventilator days	18.02 ± 16.97	39.49 ± 17.55	9.39 ± 4.54	<0.001
Hospital mortality, No. (%)	44 (17.5)	30 (41.7)	14 (7.8)	<0.001

Abbreviations: ICU, intensive care unit; OPD, outpatient department.

**Table 2:** Factors, hematology, biochemistry, FiO_2_, and PaO_2_/FiO_2_ on admission day 7.

Factors	Univariate analysis		
	Ventilator-dependent (N = 72)	Ventilator-independent (N = 179)	*p*-value
Age, years, mean ± SD	69.40 ± 15.65	64.59 ± 15.67	0.028
Age > 69 y/o	42 (58.3)	76 (42.5)	0.023
Male sex, No. (%)	42 (58.3)	111 (62.0)	0.589
APACHE II	24.80 ± 8.51	24.22 ± 9.34	0.664
Initial SOFA score	8.15 ± 3.52	8.80 ± 3.50	0.184
Charlson index	2.79 ± 2.08	2.36 ± 1.85	0.111
Underlying comorbidities, No. (%)			
oronary artery disease	22 (30.6)	35 (19.6)	0.060
Hypertension	40 (55.6)	108 (60.3)	0.486
Chronic obstructive pulmonary disease	7 (9.7)	23 (12.8)	0.490
Asthma	2 (2.8)	4 (2.2)	0.799
Cirrhosis	6 (8.3)	12 (6.7)	0.651
Diabetes mellitus	28 (38.9)	86 (48)	0.188
A history of stroke	22 (30.6)	32 (17.9)	0.027
Chronic kidney disease	21 (29.2)	40 (22.3)	0.255
Pulmonary tuberculosis	10 (5.6)	4 (5.6)	1.000
With malignancy	26 (14.5)	16 (22.2)	0.140
White blood cells, 10 × 10^3^/μl	12.03 ± 8.01	11.87 ± 5.16	0.857
Hemoglobin, g/dl	10.05 ± 1.66	10.57 ± 1.59	0.024
Platelet, 1000/μL	177.09 ± 97.17	234.66 ± 138.86	<0.001
Prothrombin time, sec	12.33 ± 1.29	12.44 ± 1.25	0.527
INR	1.19 ± 0.13	1.20 ± 0.12	0.487
AST, U/L	58.03 ± 44.31	54.13 ± 41.39	0.508
ALT, U/L	51.51 ± 39.35	64.04 ± 88.05	0.247
BUN, mg/dL	47.51 ± 37.16	38.70 ± 29.94	0.076
Creatinine, mg/dL	2.06 ± 2.17	1.95 ± 2.29	0.706
Na, meq/L	141.21 ± 6.17	138.89 ± 11.31	0.102
K, meq/L	3.89 ± 0.72	3.86 ± 0.72	0.791
C-reactive protein, mg/L	77.41 ± 77.45	55.03 ± 47.30	0.025
Albumin, g/dL	3.52 ± 3.49	4.21 ± 12.33	0.638
Lactate, mmol/L	1.52 ± 0.67	1.46 ± 0.55	0.470
Procalcitonin, ng/mL	8.17 ± 24.45	5.21 ± 8.83	0.319
pH	7.44 ± 0.67	7.46 ± 0.45	0.009
PaCO_2_, mmHg	40.92 ± 12.14	38.29 ± 6.92	0.087
Bicarbonates, mmol/L	27.21 ± 6.96	26.89 ± 4.86	0.723
FiO_2_, %	39.46 ± 11.90	35.02 ± 3.43	0.003
PaO_2_/FiO_2_, mmHg	269.46 ± 91.22	297.50 ± 83.48	0.020

Abbreviations: APACHE, Acute Physiologic and Chronic Health Evaluation; SOFA, Sequential Organ Failure Assessment; INR, International Normalized Ratio; AST, Aspartate Aminotransferase; ALT, Alanine Aminotransferase; FiO_2_, Fraction of Inspired Concentration of Oxygen.

Hematology, biochemistry, FiO_2_, and PaO_2_/FiO_2_ were collected for further analysis (Table 2). The platelet count was lower on admission day 7 in the ventilator-dependent group compared with the ventilator-independent group (*p* < 0.001). Patients with acidosis and higher FiO_2_ had an increased risk of prolonged ventilator use (*p* < 0.05).

### Ventilator dependence risk score

We constructed a ventilator dependence risk score using individual risk factors, which were first identified from the univariate analysis. Risk factors on admission day 1, day 3 and day 7 in univariate analysis included white blood cells, hemoglobin, platelet, prothrombin time, INR, AST, ALT, BUN, creatinine, Na, K, C-reactive protein, albumin, lactate, procalcitonin, pH, PaCO_2_, Bicarbonate, FiO_2_, PaO_2_/FiO_2_, etc. Variables on admission day 1, admission day 3 and admission day 7, which were possibly associated with ventilator dependence in the univariate analysis (*p* < 0.1), were included in a multivariate analysis model. A total of 11 variables were included in the multivariate analysis model, such as age >69 years, FiO_2_ >39%, history of stroke, pH < 7.35, platelet <150,000/μL, history of coronary artery disease, hemoglobin, BUN, C-reactive protein, PaO_2_/FiO_2_ and PaCO_2_ on admission day 7. After stepwise method, the independent factors associated with ventilator dependence were identified to build a score. A clinical score (VD risk score) was calculated based on four variables independently associated with ventilator dependence in the multivariate analysis, including previous stroke, thrombocytopenia, acidosis, and higher FiO_2_ (Table 3). The ventilator dependence risk score was calculated as the sum of these four variables after adjusting by proportion to beta coefficient. We assigned a history of stroke one point, platelet count on admission day 7 of less than 150,000/μL one point, pH value on admission day 7 of less than 7.35 two points, and the fraction of inspired oxygen on admission day 7 over 39% two points (Table 4).

**Table 3:** Factors on admission day 7 for patients with sepsis and septic shock after multivariate analysis.

Multivariate analysis				
Factors	Coefficient	OR	95% C.I.	*p*-value
Previous stroke	0.941	2.564	1.287–5.106	0.007
Platelet ≤ 150,000/μL	0.946	2.575	1.383–4.795	0.003
pH ≤ 7.35	1.580	4.855	1.107–21.290	0.036
FiO_2_ ≥ 39%	1.658	5.248	2.543–10.834	<0.001

Abbreviations: OR, odds ratio; C.I, confidence interval.

**Table 4:** Variables included in ventilator dependence risk score.

Variables for ventilator dependence risk score	Value	Points
Previous stroke	Previous stroke event	1
Platelet amount on admission day 7	≤150,000/μL	1
pH value on admission day 7	≤7.35	2
FiO_2_ on admission day 7	≥39%	2
VD risk score = previous stroke + Plt 7 + 2*pH 7 + 2*FiO_2_ 7	Score range	0–6

Abbreviations: FiO_2_, Fraction of Inspired Oxygen; VD risk score, Ventilator Dependence Risk Score.

**Figure 3:**
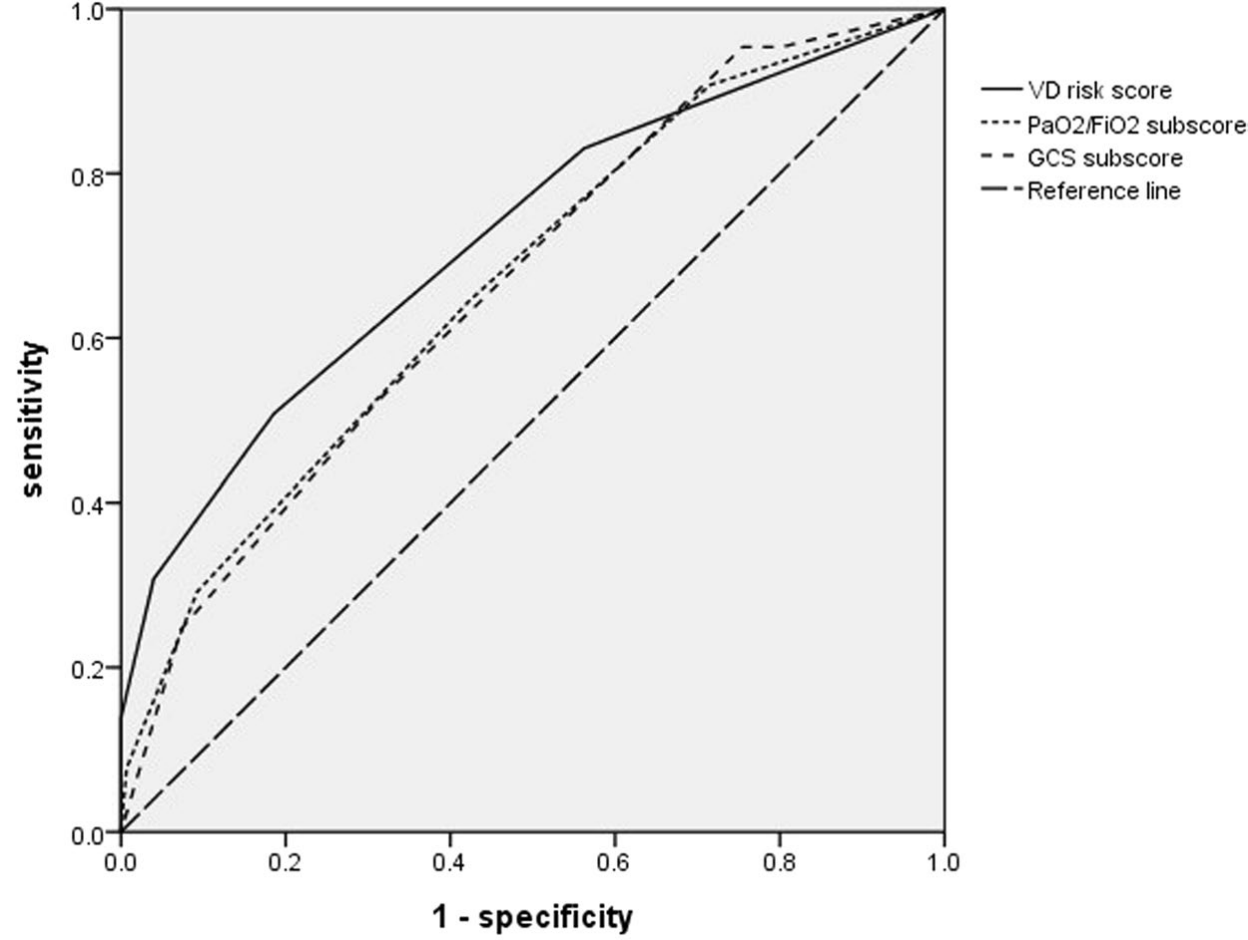
Ventilator dependence risk score compared to PaO_2_/FiO_2_ subscore and GCS subscore on admission day 7.

Receiver operating characteristic curves were plotted in Fig. 3. The area under the curve (AUC) of the ventilator dependence risk score was 0.725 with *p* value < 0.001. A ventilator dependence risk score equal to or more than one point yielded 80.5% sensitivity and 50.2% specificity.

### SOFA score for ventilator dependence prediction

We tested Sequential Organ Failure Assessment (SOFA) score^19^ on admission day 1 and day 7 to predict ventilator dependence on sepsis and septic shock patients with respiratory failure. The *area under the curve (*AUC) of the SOFA score on admission day 1 was 0.441 and the AUC of the SOFA score on admission day 7 was 0.662. We also tested and found that SOFA PaO_2_/FiO_2_ subscore and GCS subscore on admission day 7 could help predict ventilator dependence on sepsis and septic shock patients with significant difference in univariate analysis (Table 5). The area under the curve of the PaO_2_/FiO_2_ subscore on admission day 7 was 0.668 and the AUC of the GCS subscore on admission day 7 was 0.673 (Fig. 3).

**Table 5:** SOFA score and subscore on admission day 7 in 251 sepsis and septic shock patients.

Factors	Ventilator-dependent (N = 72)	Ventilator-independent (N = 179)	*p*- value
SOFA score on admission day 7	6.82 ± 3.17	5.04 ± 2.95	<0.001
PaO_2_/FiO_2_ subscore	1.92 ± 1.08	1.24 ± 0.98	<0.001
Platelet subscore	0.87 ± 1.07	0.57 ± 1.00	0.05
Bilirubin subscore	0.25 ± 0.68	0.38 ± 0.79	0.265
Cardiovascular subscore	0.34 ± 0.71	0.24 ± 0.59	0.241
Glasgow coma scale subscore	2.67 ± 0.99	1.91 ± 1.18	<0.001
Creatinine or urine output subscore	1.07 ± 1.36	0.84 ± 1.41	0.252

### Validation ventilator dependence risk score

**Figure 4:**
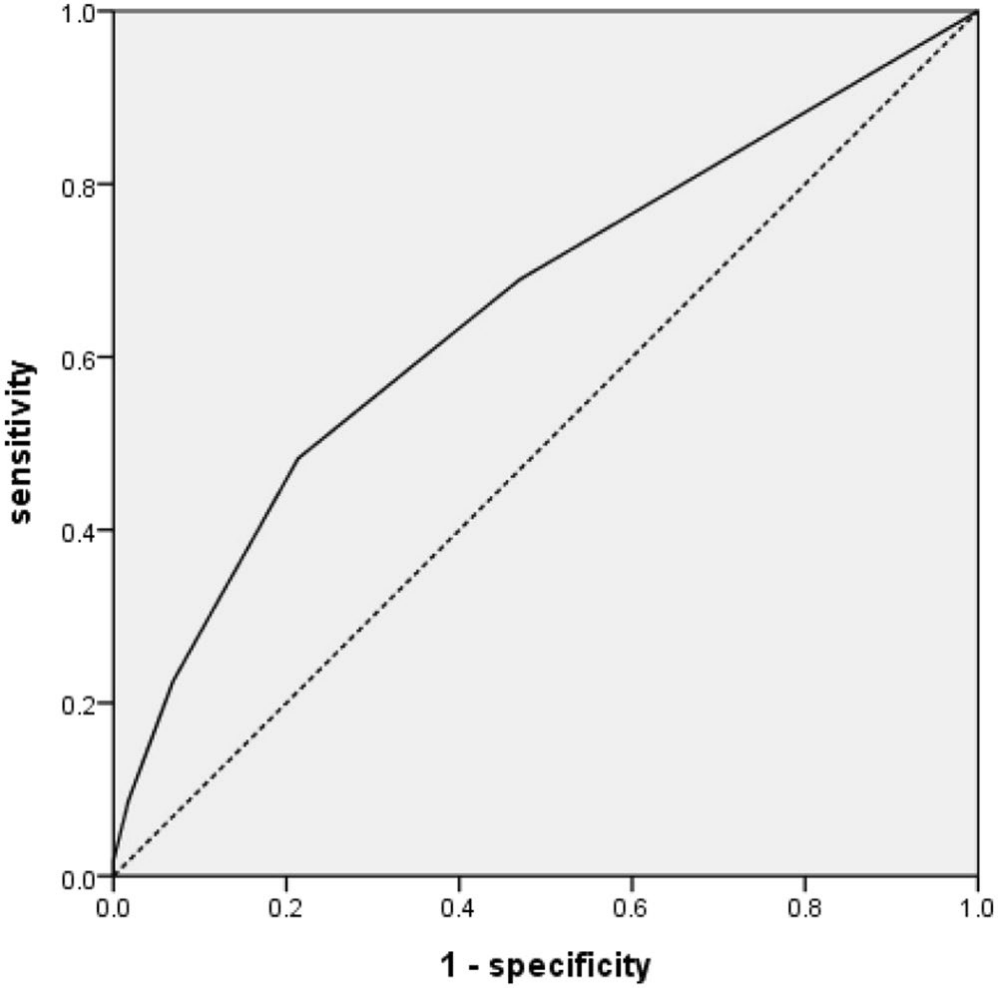
Receiver operating characteristic curve of the ventilator dependence risk score for discrimination between patients with ventilator dependence and ventilator independence in the ICU in the validation group of 175 patients with sepsis/septic shock and respiratory failure.

**Figure 5:**
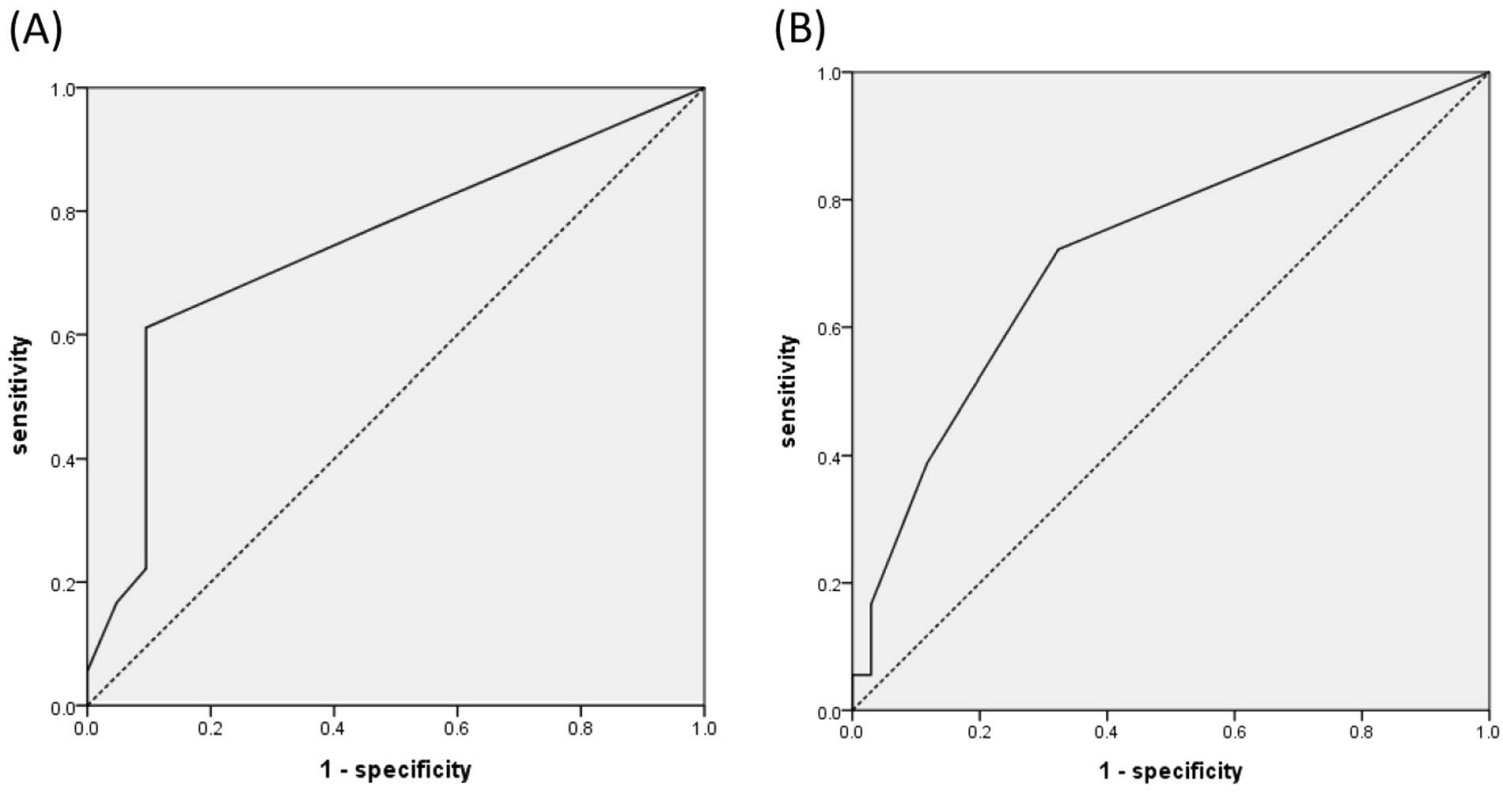
(**A**) Ventilator dependence risk score in sepsis and septic shock patients in the cancer group revealed AUC = 0.745. (**B**) Ventilator dependence risk score in sepsis and septic shock patients in the chronic kidney disease group revealed AUC = 0.723.

For validation, we collected data from sepsis/septic shock patients with mechanical ventilation admitted to the medical ICU from November 2015 to November 2016. We used the ventilator dependence risk score to predict ventilator dependence in these 175 patients. Patient characteristics and underlying disease are collected in revealed as Table 6. The AUC of the ventilator dependence risk score was 0.658 and the *p*-value was 0.001 (Fig. 4). A ventilator dependence risk score equal to or more than one point yielded 69.0% sensitivity and 53.0% specificity. We also found the AUC of ventilator dependence risk score was 0.745 in the cancer group and *p*-value was 0.009. AUC of ventilator dependence risk score was 0.723 in the chronic kidney disease group (p = 0.009) (Fig. 5).

**Table 6:** Patient characteristics on validation group (N = 175).

Factors	Univariate analysis		
	Ventilator-dependent (N = 58)	Ventilator-independent (N = 117)	*p*-value
Age, years, mean ± SD	70.24 ± 12.49	64.47 ± 14.37	0.010
Age > 69 y/o	33 (56.9)	50 (42.7)	0.077
Male sex, No. (%)	33 (56.9)	64 (54.7)	0.783
APACHE II	23.84 ± 9.78	22.48 ± 8.59	0.386
Initial SOFA score	7.97 ± 2.66	6.97 ± 3.20	0.042
Charlson index	2.39 ± 2.11	3.02 ± 2.25	0.097
Underlying comorbidities, No. (%)			
Coronary artery disease	17 (29.3)	30 (25.6)	0.606
Hypertension	32 (55.2)	60 (51.3)	0.628
Chronic obstructive pulmonary disease	16 (27.6)	14 (12.0)	0.010
Asthma	1 (1.7)	1 (0.9)	0.610
Cirrhosis	3 (5.2)	8 (6.8)	0.669
Diabetes mellitus	28 (48.3)	51 (43.6)	0.558
Previous stroke	12 (20.7)	14 (12.0)	0.127
Chronic kidney disease	18 (31.0)	34 (29.1)	0.788
Pulmonary tuberculosis	5 (8.6)	7 (6.0)	0.516
With malignancy	18 (31.0)	21 (17.9)	0.121

## Discussion

In this study, we identified risk factors for prolonged mechanical ventilation in patients who survived sepsis and septic shock. These included a history of stroke, and data collected on day 7 (thrombocytopenia, acidosis, and a higher fraction of inspired oxygen). The ventilator dependence risk score can help easily predict prolonged mechanical ventilation. We chose biochemical and physiological variables from day 7 to incorporate into our score, as opposed to day 1 or day 21, which would each have advantages and disadvantages. For example, it is too late to predict ventilator dependency using day 21 data. On the other hand, with multiple factors and different treatment response, it is difficult to predict ventilator dependency from day 1 data. With aggressive treatment in the first week, day 7 data can help identify which patients face a substantial risk of becoming long-term ventilator-dependent.

Patients who have suffered a stroke in the past often have respiratory dysfunction due to respiratory drive impairment. According to a previous study, respiratory function depends on numerous neurologic structures, which extend from the cerebral cortex to the medulla; complications after an injury to the respiratory center could lead to prolonged mechanical ventilation^20,21^. Therefore, a previous stroke is an independent risk factor for predicting prolonged ventilator use.

Sepsis is a life-threatening organ dysfunction caused by a disproportionate host response to infection and it involves complex mechanisms^22^. During sepsis, platelet numbers decrease due to increased platelet destruction. Sepsis may result in hypercoagulation due to fibrin deposition and platelet activation. This leads to the formation of micro-thrombi as a host defense mechanism against pathogens, in which platelets play a crucial role. In extreme situations, this may progress to disseminated intravascular coagulation (DIC), with severe thrombocytopenia and coagulation system impairment^23–25^. Platelet dysfunction during sepsis correlates with a poorer prognosis. Thus, the morphology, number, and function of platelets may be used as biomarkers for the risk stratification of patients with sepsis^25^. Although we excluded very ill patients with decreased platelet counts who expired within 21 days (in our series, average 152*10^3^/μL), the platelet count on day 7 could differentiate the ventilator-dependent and independent groups on day 21. A relatively low platelet count on admission day 7 suggests that a septic patient has not completely recovered and may have greater risk of ventilator dependence. Although there was a significantly lower hemoglobin level in the ventilator-dependent group, it is hard to suggest that bleeding caused by thrombocytopenia is causing weaning failure. The hemoglobin level in both groups was greater than 10 g/dl.

Acidosis is increased acidity (hydrogen ion concentration) in the blood and other body tissues. It occurs when the arterial pH falls below 7.35. Sepsis can cause tissue hypoperfusion and the accumulation of lactate, which causes metabolic acidosis^26^. Acidosis resolution in survivors was attributable to a decrease in strong ion gap and lactate levels^26^. Additionally, respiratory acidosis can be due to the accumulation of carbon dioxide in the lungs, which indicates poor lung functioning^27^. Our data revealed that arterial blood gas acidosis on day 7 was one of the independent risk factors predicting ventilator dependence. We did not find statistical differences between groups on higher lactate levels or vasopressor use trends in ventilator dependent patients. Acidosis could be non gap metabolic acidosis from hyperchloremia and fluid overload. In addition, either sepsis progression or poor lung functioning may have caused the resulting acidosis. Fraction of inspired oxygen (FiO_2_) is the fraction or percentage of oxygen in the volume being measured. It is used to represent the percentage of oxygen participating in gas exchange. According to a study by Diniz *et al*., FiO_2_ levels sufficient to ensure a SpO_2_ ≥92% do not alter breathing patterns or trigger clinical changes in weaning patients^28^. The FiO_2_ level was enough to represent the oxygen status of the ventilated patient. Our data revealed that a higher fraction of inspired oxygen demand was associated with greater risk of ventilator dependence in patients with sepsis or septic shock.

Applying the ventilator dependence risk score to predict prolonged ventilator dependence can help us communicate with the family, enable quick adjustment of the treatment strategy, and ensure more efficient allocation of medical resources. In addition, it is clinically applicable. The score includes two components. One component is uncorrectable, such as previous stroke history; the other component is correctable if treatment is successful, such as thrombocytopenia, acidosis, and fraction of inspired oxygen. We do not suggest correction of thrombocytopenia and acidosis by blood transfusion and bicarbonate use, as there are inherent risks with platelet transfusion and bicarbonate infusion. However, the clinical physician should make the best efforts in correcting underlying progressive sepsis to avoid prolonged ventilator use. We do not routinely use subcutaneous heparin for prophylaxis of deep vein thrombosis or pulmonary embolism in Taiwan. Therefore, we seldom have heparin induced thrombocytopenia patients. In our study group, we had no patients with sepsis and pulmonary embolism concurrently. However, we should keep the possibility in mind.

As some components of our ventilator dependency risk score are similar to SOFA values, we tested SOFA score for ventilator dependence prediction. We found the area under the curve (AUC) of the ventilator dependence risk score (0.725) was better than the SOFA score on admission day 1 and day 7. However, 2 components of SOFA score (pulmonary subscore: PaO_2_/FiO_2_ and GCS subscore) on admission day 7 were significant for predicting ventilator dependence in univariate analysis (p < 0.001). Despite these findings, the PaO_2_/FiO_2_ and GCS AUC were not better than the ventilator dependence risk score AUC (Fig. 3). In fact, we have previously described an immune dysfunction scoring system for predicting 28-day mortality in septic patients, with better discrimination than SOFA score; this system was valid and reproducible. The above cases were from part of the current sepsis cohort, who agreed for immune function assessment^29^. However, in the present study, we are focused on ventilator dependency amongst patients who survive sepsis more than 21 days. Combining those 2 tools, we can predict long term ventilator dependence and predict survival.

The area under the curve (AUC) of the ventilator dependence risk score was 0.725 in our study group and the AUC of the ventilator dependence risk score was 0.658 in the validation group. After further analysis of the validation group, we found the AUC of ventilator dependence risk score was 0.745 for sepsis with cancer group and the AUC was 0.723 for sepsis with chronic kidney disease group. We are actively studying the effect of co-morbidity on the outcomes of patients with sepsis, although it is out of the scope of this study. Our previous study revealed that among patients admitted to the ICU with sepsis, those with underlying active cancer had higher baseline levels of plasma IL-10, higher trend of G-CSF and higher mortality rate than those without active cancer^30^. Our ventilator dependence risk score could help predict who will need prolonged mechanical ventilation. We did not exclude patients with tuberculosis or severe immunosuppression (human immunodeficiency virus (HIV), oncologic, solid-organ or bone marrow transplantation). Our score can also be used for these patients.

Septic patients admitted to the hospital or the intensive care unit are usually screened for contamination with multi-resistant bacteria and subjected to collection of blood cultures and respiratory secretions. As in our previous study^31^, multi-resistant bacteria or specific pathogens influence survival in patients with ventilator associated pneumonia. The phenomenon was not shown for ventilator dependency^14^. Most of our patients came from ER (69.3%) and most of our blood culture showed no growth. We suggest that multi-resistant bacteria may not influence prediction of prolonged mechanical ventilation. However, further study may be needed to determine the effect.

Renal replacement therapy could be a risk factor. However, there was no statistical significance in univariate analysis. In addition, the SOFA renal subscores did not differ between ventilator dependent and independent patients. Therefore, renal replacement therapy was not used in the scoring system.

In 2011, Sellares J *et al*.^32^ described that COPD, increased heart rate and PaCO_2_ during the spontaneous breathing trial independently predicted prolonged weaning. However, our studied group had small proportion of COPD (9.7% in ventilator-dependent group and 12.8% in ventilator-independent group) (Table 2). In addition, we did not routinely record heart rate and PaCO_2_ during the spontaneous breathing trial. Therefore, PaCO_2_ and heart rate during the spontaneous breathing trial were not included in our scoring model. Extubation failure before day 7 may be an additional prognostic parameter for ventilator dependence. However, in our study population, no extubation failure before day 7 was noted.

The limitations of the study include the retrospective study design and possible selection bias. However, first, we used prospectively collected data and screened consecutive patients. Second, we excluded patients who died within 21 days, which may have masked some predictors associated with both mortality and ventilator dependence. However, mortality prediction was beyond the scope of this study. The application of the score focused on patients who survived sepsis/septic shock with acute respiratory failure on admission day 7. This patient group was not completely recovered and needed further treatment and strategic decision making. From our results, the data from day 7 is enough to calculate the score, which makes it feasible to use for predicting ventilator dependence. Patients require mechanical ventilation due to either pulmonary function problems or neurological function problems. In patients with sepsis, both components may co-exist. It is difficult to delineate what proportion of patients requiring prolonged mechanical ventilation is attributed to pulmonary or neurological problems. We did not incorporate any data on the patients’ pulmonary system mechanics or respiratory muscle strength (respiratory system compliance or resistance, maximal exhaled tidal volume, negative inspiratory force, rapid shallow breathing index) that are typically studied during weaning from mechanical ventilation^33^. It is partially because of some missing data owing to the retrospective characteristic of the study, which makes it difficult to analyze. Most importantly, obtaining parameters like static compliance requires an additional procedure such as paralysis and muscle relaxant, which may add risk to those patients with unstable severe sepsis. For easy application to patients with sepsis and septic shock, we chose to incorporate data easily checked in clinical practice.

It is now well known that sepsis and multi organ failure can cause neurological dysfunction by way of critical illness neuropathy and myopathy (i.e., ICU acquired weakness), which can cause difficulty weaning from mechanical ventilation due to diaphragmatic weakness. Sepsis and multiple organ dysfunction are the most common and well accepted risk factors for ICU acquired weakness. Some other risk factors like ARDS, neuromuscular blockade, glucose control, and steroid use are missing from the analysis due to the retrospective study design. Those particular entities deserve attention. The diagnosis of ICU acquired weakness is often clinical with EMG support, which is not often conducted in routine clinical practice.

With respect to neurological function, we note a significant difference in the groups with the history of prior stroke. We did not have complete data differentiating hemorrhagic or ischemic strokes. In addition, the functional status or delirium data were also lacking. We attempted to use GCS (the required data are already present within the APACHE and SOFA scores) but the results showed poor discrimination. Those issues need to be explored further in the future.

A valuable tool to predict which septic patients will need prolonged mechanical ventilation may have not only therapeutic ramifications, but also significant financial and social implications. As shown in Table 1, patients requiring long term mechanical ventilation have significantly longer ICU stay and in hospital mortality. It is primarily due to the medical acuity. However, in part, it is also due to a paucity of ventilator weaning facilities. Patients who require long term mechanical ventilation are often difficult to place, leading to longer hospital stays than expected for their given illness.

We did not discuss diagnosis of ARDS in this study. The PaO_2_/FiO_2_ were comparable between the two groups. In the same period, our colleagues participated in a multiple center study showing the effects of ARDS and fluid balance on outcomes. Over resuscitation leads to fluid overload and pulmonary edema, and hypoxia, which may influence ventilator dependence. We found a negative day 1–4 cumulative fluid balance was associated with a lower mortality rate in critically ill patients with influenza^34^. We are now exploring whether cumulative fluid balance predicts ventilator dependency. We need to further evaluate an association between over resuscitation and ventilator dependence in the future.

Ventilator dependence risk score, including a history of stroke and data from day 7 (thrombocytopenia, acidosis, and the higher fraction of inspired oxygen), can be applied to predict prolonged mechanical ventilation in patients who survive sepsis and septic shock.

## Materials and Methods

### Setting and study design

This retrospective study was conducted in three medical ICUs, including 34 beds at Kaohsiung Chang Gung Memorial Hospital, a 2,700-bed tertiary teaching hospital in southern Taiwan. Consecutive adult patients (aged ≥18 years) with acute respiratory failure on admission to the medical ICU with sepsis/septic shock were surveyed from August 2013 to October 2015 through chart review. We excluded patients who passed away within 21 days, those whose families requested palliative treatment before day 21, and those who were already long-term mechanical ventilator dependent. The enrolled patients were divided into two groups, i.e., ventilator-independent and ventilator-dependent, according to their ventilator status at the time of ventilator use on day 21 from the chart review (Fig. 1). We also collected data from sepsis/septic shock patients with respiratory failure who were admitted to the medical ICU from November 2015 to November 2016 as the validation group (Fig. 2).

The study was approved by the Institutional Review Board (IRB) of Chang Gung Memorial Hospital and the requirements to obtain informed consent from patients were waived by IRB (105–6824C). We confirmed that all methods were performed in accordance with the relevant guidelines and regulations.

### Definitions

Long-term ventilator dependence in patients was defined as the need for mechanical ventilation for more than six hours per day for more than 21 days^16^. Sepsis was defined as a life-threatening organ dysfunction due to a disproportionate host response to infection^35^. Patients with septic shock were identified by a vasopressor requirement to maintain a mean arterial pressure of >65 mmHg in the clinical condition^36,37^. All enrolled patients met the new criteria for sepsis and required mechanical ventilation at the time of admission to the ICU. Moreover, they survived for at least 21 days after admission to the ICU. IV titratable sedation was applied if the patient’s condition required and the titration protocol was standardized by medical intensive care unit. The standard clinical practices for weaning the patient from mechanical ventilation were performed during the study period (i.e., pressure support and spontaneous breathing trials).

### Data collection

Clinical data were retrieved from medical records and included age, gender, Sequential Organ Failure Assessment (SOFA) score^19^, Acute Physiological Assessment and Chronic Health Evaluation II (APACHE II) score^38^, Charlson Index and underlying comorbidities^39,40^, and other clinical factors possibly related to prolonged ventilator use. We also collected hematology, biochemistry, fraction of inspired oxygen (FiO_2_) and PaO_2_/FiO_2_ on admission day 7 to follow up on the patient’s condition. All variables were evaluated as possible risk factors of prolonged ventilator use.

### Score construction and calculation

Categorical variables were analyzed using the chi-squared test, and continuous variables were compared using the Student’s *t*-test. A two-tailed P value of <0.05 was considered to indicate a significant result. Univariate analysis was used to identify significant risk factors associated with ventilator dependence. Variables associated with ventilator dependence in the univariate analysis (*p* < 0.1) were included in a multivariate analysis model. Using the stepwise method, the independent factors associated with ventilator dependence were identified to build a score using the Hosmer-Lemeshow goodness-of-fit test. A clinical score (VD risk score) was calculated based on four variables independently associated with ventilator dependence in the multivariate analysis. The number of points assigned to each variable in the VD score was adjusted according to proportion to beta coefficient in the regression model. The VD risk score is the sum of the points for these four variables. The receiver operating characteristic (ROC) curve was used to evaluate the performance of the VD risk score.

All statistical analysis was performed using the SPSS 22.0 software package (SPSS Inc., Chicago, IL, USA).
